# High-yield fabrication and properties of 1.4 nm nanodiamonds with narrow size distribution

**DOI:** 10.1038/srep38419

**Published:** 2016-12-02

**Authors:** Stepan Stehlik, Marian Varga, Martin Ledinsky, Daria Miliaieva, Halyna Kozak, Viera Skakalova, Clemens Mangler, Timothy J. Pennycook, Jannik C. Meyer, Alexander Kromka, Bohuslav Rezek

**Affiliations:** 1 Institute of Physics ASCR, Cukrovarnická 10, Prague, 162 00, Czech Republic; 2 Faculty of Electrical Engineering, Czech Technical University in Prague, Technická 2, Prague, 16627, Czech Republic; 3 Physics of Nanostructured Materials, Faculty of Physics, University of Vienna, Boltzmanngasse 5, 1090 Vienna, Austria

## Abstract

Detonation nanodiamonds (DNDs) with a typical size of 5 nm have attracted broad interest in science and technology. Further size reduction of DNDs would bring these nanoparticles to the molecular-size level and open new prospects for research and applications in various fields, ranging from quantum physics to biomedicine. Here we show a controllable size reduction of the DND mean size down to 1.4 nm without significant particle loss and with additional disintegration of DND core agglutinates by air annealing, leading to a significantly narrowed size distribution (±0.7 nm). This process is scalable to large quantities. Such molecular-sized DNDs keep their diamond structure and characteristic DND features as shown by Raman spectroscopy, infrared spectroscopy, STEM and EELS. The size of 1 nm is identified as a limit, below which the DNDs become amorphous.

Nanodiamond brings the extraordinary properties of diamond down to the nanometer scale and thereby opens prospects for exploiting structural, electronic and optical effects related with large surface-to-volume ratios and size-confinement in diamond^1^,^2^,^3^,^4^. Biocompatibility, widely adjustable surface chemistry, and photoluminescence features make nanodiamond promising for applications ranging from quantum physics to biomedicine^5^,^6^,^7^,^8^,^9^,^10^. Since its discovery in the sixties of the last century detonation nanodiamonds (DNDs) have attracted increasing attention from both scientific and applications perspectives^11^. Successful preparation of colloidal solutions containing truly individual 4–5 nm DND nanoparticles^12^,^13^,^14^ enabled the construction of a model of an idealized DND nanoparticle^15^. According to this model, a typical DND nanoparticle is composed of: (1) a crystalline diamond core formed by sp^3^ hybridized carbon atoms, (2) a disordered transient sp^3^/sp^2^ layer, (3) a surface shell that accommodates various surface functional groups which saturate dangling bonds and are responsible for colloidal and chemical properties of DNDs. Such complex DND structure is for instance reflected by specific features in Raman spectrum^3^,^16^,^17^.

An extreme reduction of size of nanodiamonds to below 2 nm would enable exploration of a new area between chemistry and nanotechnology. For instance, Vlasov *et al*. showed^2^ that NDs of meteoritic origin as small as 1.6 nm are capable of housing stable photoluminescent silicon vacancy (SiV) colour centres^18^. Such molecular-sized fluorescent nanodiamonds might be used as ultrasmall non-perturbative fluorescent markers and probes in microscopy and sensing. However colour centres-related luminescent or semiconducting properties of artificial nanodiamonds depends on doping the nanodiamonds^19^ via desired elements such as nitrogen^20^ or boron^21^. Availability of sub-2 nm nanodiamonds would also enable exploration of quantum phenomena in diamond^22^. These are still poorly understood in contrast to e.g. Si where nanoscale dimensions of Si nanocrystals together with a tensile stress induced by proper surface passivation may convert the Si nanocrystal to a luminescent material with a direct bandgap^23^. Bolker *et al*.^24^ recently demonstrated quantum confinement effects in laser-synthesized nanodiamonds by scanning tunneling spectroscopy for nanodiamonds smaller than 4.5 nm. However deeper understanding of quantum phenomena as well as fundamental experimental data on sub-2 nm nanodiamonds is currently missing.

In order to fully exploit this potential, the size of synthetically produced nanodiamonds must be reduced, from the presently commercially available 5 nm and above, down to 1 nm. Several bottom-up^25^ as well as top-down^26^,^27^ approaches were reported to reduce the size of nanodiamonds but none of these approaches reached mean sizes below 2 nm. Thermal annealing in air is often used to purify the NDs, removing sp^2^ carbon^28^,^29^, but it may also, in principle, be used for size reduction of NDs through the “burning” of the diamond itself. Indeed, the size reduction of high-pressure high-temperature (HPHT) nanodiamonds by thermal annealing was recently demonstrated^3^,^30^,^31^. Carefully air-annealed HPHT NDs were stable down to 1.1 nm and remained fully crystalline without any apparent non-diamond shell at this small size confirming the nanodiamond stability down to 1 nm^3^. However, such small HPHT NDs were present only in small amounts and did not represent a major fraction of the final material. A great advantage of DNDs is an inherent narrow size distribution (typically 5 ± 2 nm) in comparison with broad distribution of HPHT NDs^32^. This may potentially enable reaching relatively high yields of DNDs with reduced sizes. The feasibility of such a process was recently indicated by a study on the thermal annealing of isolated DNDs on a substrate^33^.

Here we show that annealing in air under specific conditions can be used for extreme yet controllable reduction of the DNDs mean size well below 2 nm on a bulk scale. In addition, the process promotes efficient disaggregation of DND powder, including core agglutinates, which increases the yield of individual DND particles. UV-Raman spectroscopy, scanning transmission electron microscopy (STEM), and Fourier transform infrared spectroscopy (FTIR) evidence consistent nanodiamond quality and nanodiamond surface chemistry throughout the size reduction process while the sp^2^ content is significantly reduced at the same time.

## Results and Discussion

A suitable annealing temperature for controllable size reduction of DNDs was estimated from thermogravimetric analysis (TGA)^3^. We selected a temperature of 520 °C as it lies between the onset of DND thermal weight loss (480 °C) and the point where intensive weight loss starts (560 °C). We suppose that the weight loss during the annealing at 520 °C (Supplementary Fig. S1) is due to oxidative etching of the DNDs, *i.e.* carbon is oxidized by oxygen in air to carbon dioxide, which must be accompanied by decrease of the DNDs size.

**Figure 1:**
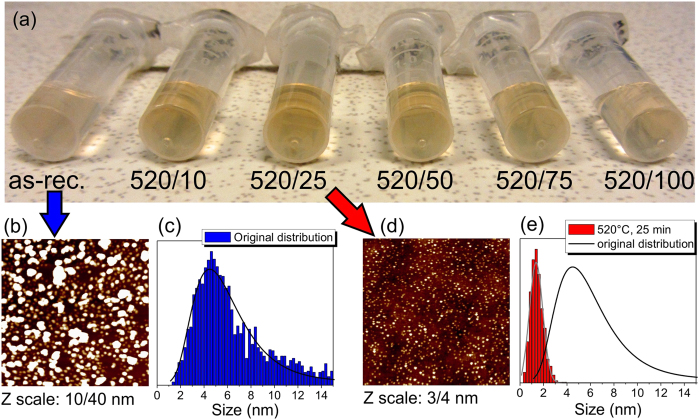
DND colloids and related atomic force microscopy (AFM) images/size distribution data. (**a**), Photograph of the colloidal solutions prepared from an identical amount of DND powder (3 mg). Annealing temperature/time data are given below each particular vial. (**b**,**c**), AFM images of the as-received DNDs and the corresponding size distribution histogram. (**d**,**e**), AFM images of the DNDs annealed at 520 °C for 25 min their size distribution histogram. The scan area is 1 × 1 μm^2^ for both images. For better contrast the Z scales are adjusted to 10 nm from 40 nm (**b**) and to 3 nm from 4 nm (**d**).

Figure 1a shows a photograph of the prepared DND colloidal solutions prior and after the annealing in air. All the colloidal solutions were prepared using 3 mg of DND powder with a subsequent 1 h ultrasonication, and 1 h centrifugation to be visually directly comparable. It is noticeable that all the solutions prepared from the annealed DND powder look more concentrated (more coloured) than the solution prepared from the as-received DND powder. Moreover, the solution obtained from the DND powder annealed at 520 °C for 25 min looks the most concentrated. In order to determine the DND weight and particle concentration in the colloids we performed a weight analysis of the DND residual sediment after the sonication-centrifugation treatment using 10 mg of the as-received DND powder and the DND powder annealed at 520 °C for 25 min. For both samples, we found that approximately 30 wt. % of the total amount of DNDs was dispersed in supernatant. This value corresponds well to 40 wt.% reported by Osawa *et al*.^34^ by using a more powerful 400 W ultrasonic processor. The concentration of the colloids shown in Fig. 1a is thus estimated to be ~0.5 mg/mL if we take the 30 wt. % dispersibility into account.

A typical 1 × 1 μm^2^ AFM image and derived size distribution histogram of the as**-**received DNDs deposited from the related colloid are shown in Fig. 1b and c respectively. The size distribution fit of the as-received DND has a maximum at 4.6 nm corresponding to individual DND particles and a tail ranging up to 40 nm due to presence of core agglutinates^35^ in which the DNDs are bound together by strong facet-facet type electrostatic interactions^36^. In contrast, the AFM image and corresponding size distribution histogram of DNDs annealed at 520 °C for 25 min (Fig. 1d,e) show that the mean size value is reduced down to 1.4 nm. In addition, the vast majority of the annealed DNDs have sizes below 3 nm. The width of the size distributions decreased from ±2.5 nm for the as-received DNDs down to ±0.7 nm after annealing for 25 min as derived from full width at half maximum (FWHM) values of the histogram fits. This indicates that the boundaries between the particular DNDs in the core agglutinates are preferentially attacked by oxygen in the air, which apart from the size reduction leads to efficient disintegration of the core agglutinates. This represents a positive side effect of the air annealing at 520 °C, primarily intended for size reduction of DNDs. The yield of the process taking into account the sonication and centrifugation applied can be roughly estimated as follows: from e.g. 10 mg of as-received DND powder one gets 3 mg (30% dispersibility), i.e. ~1.7 × 10^16^ of 4.6 nm DND particles. When air annealing at 520 °C for 25 min is applied to 10 mg of the starting DND powder one gets approx. 1.2 mg (30% dispersibility, ~40% residual mass (Fig. S1)) which corresponds to 2.4 × 10^17^ of 1.4 nm DND particles. Then the yield is approximately 1430%. In addition, the size reduction of the DNDs is not accompanied by any significant change in the surface chemistry in comparison to only surface oxidized DNDs at 450 °C for 30 min. This means that surface of the size-reduced DNDs is terminated by oxygen containing functional groups such as anhydrides and lactones. For details, see the FTIR spectra in supplementary information (Supplementary Fig. S2).

**Figure 2:**
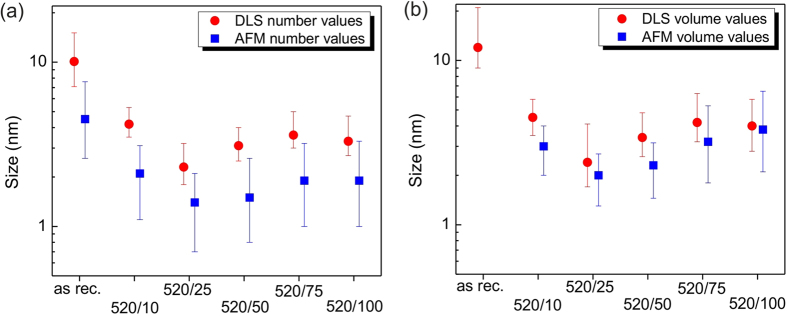
AFM and dynamic light scattering (DLS) size distribution data of the annealed DNDs. Comparison of the mean size of the DNDs as a function of the annealing duration (0–100 min at 520 °C) as obtained from DLS (red) and AFM (blue) experiments and plotted as a number distribution (**a**) and a volume distribution (**b**) in a semi-logarithmic scale. The error bars represent the width of the size distribution given by the FWHM values. The small x-offset between the DLS and the AFM data is just for a better visual clarity.

Figure 2 shows a comparison of DNDs mean sizes before and after annealing, obtained from DLS and AFM techniques as a particle number distribution (Fig. 2a) and a particle volume distribution (Fig. 2b). The error bars represent FWHM values obtained from Gaussian or lognormal fits. The asymmetry of the lognormal distribution is taken into account and reflected in the related data points. Using both AFM and DLS as for size measurement based on different principles appears very beneficial since both techniques reveal interesting facts about the annealed DNDs. First of all, both DLS and AFM data clearly follow the same trend, *i.e.* both techniques show considerable reduction of the DND mean size with a distinct minimum at 1.4 nm (AFM) and 2.3 nm (DLS) for the annealing time of 25 min. This indicates that there is no selective deposition of the DNDs on the Si substrates from the colloids. Yet, the mean size values obtained from DLS in the number distributions (Fig. 2a) are higher than those from the AFM analysis. This discrepancy may have several reasons, but we suppose that the main effect comes from a hydration shell formed in water on oxidized DND surface. Such a shell of surface-bound water on DNDs^37^,^38^ was reported to have thickness of about 0.6 nm^39^. This value agrees well with our results as the average difference between the AFM and DLS results is 1.6 ± 0.2 nm which yields a hydration shell thickness of 0.8 ± 0.1 nm. In addition to the effect of a hydration shell, other aspects like the refractive index of diamond and the viscosity of water should also be considered since they are involved in the DLS fitting model. For DND nanoparticles, the refractive index may slightly deviate from that of bulk diamond due to their complex structure and also because the viscosity of the colloidal solution may deviate from that of water^40^. This is why we consider the AFM data as more accurate and statistically relevant.

Interestingly, the mean size exhibits a minimum (1.4 nm by AFM) after annealing for 25 min. For longer annealing times the mean size slightly increases although it still remains below 2 nm. In addition, the distribution changes from symmetric Gaussian (10, 25 min) to asymmetric lognormal (50–100 min) with increasing statistical contribution of larger particles. We suppose that this effect is due to a gradual disappearance of the smaller DNDs after >25 min annealing, thereby shifting the size distribution towards larger sizes. In other words, we observe only a size decrease of a majority of DNDs up to 25 min annealing at 520 °C without noticeable DNDs loss (optical images in Fig. 1a indicates that concentration of dispersed individual DNDs actually increases), but the longer annealing time leads to some loss of individual DNDs from this main fraction (as again supported by less coloured optical images in the Fig. 1a). The number-weighted and volume-weighted AFM size distribution histograms for all the samples are shown in Fig. S3.

**Figure 3:**
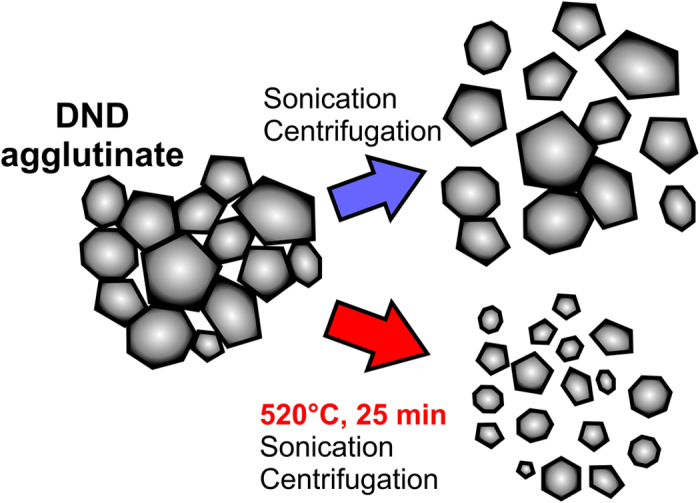
Model of DND size reduction. Schematic model showing only partial disintegration of a DND core agglutinate after sonication of the as-received DNDs. Prior application of air annealing at 520 °C for 25 min leads to enhanced disruption of the agglutinate as well as size decrease below 2 nm.

Based on the obtained results and discussion above we propose a model describing the size decrease process based on air annealing at 520 °C. The model is schematically illustrated in Fig. 3. Briefly, ultrasonication and centrifugation of the DND powder without application of the air annealing leads to only partial disintegration of the core agglutinates without affecting the DND size. When the air annealing at 520 °C is applied we observe more efficient disintegration of the core agglutinates as well as the mean size reduction well below 2 nm.

**Figure 4:**
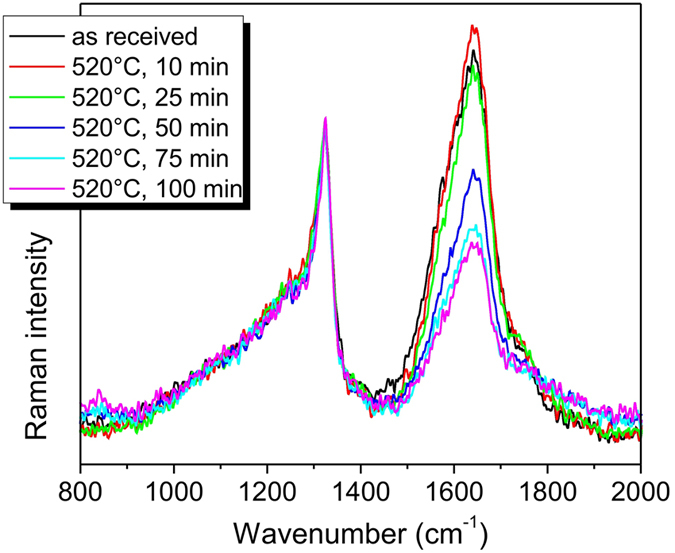
Raman spectra of the size reduced DNDs. Raman spectra of as-received DNDs (black) and the annealed DNDs at 520 °C for 10 min (red), 25 min (green), 50 min (blue), 75 min (cyan), and 100 min (magenta).

Raman spectroscopy analysis of the DNDs is shown in Fig. 4. Raman spectra of the as-received as well as size-reduced DNDs are dominated by the peak at ~1324 cm^−1^ assigned to diamond (1332 cm^−1^ in bulk). This clearly demonstrates the nanodiamond character of the DNDs down to 2 nm when volumetric AFM size distribution (Fig. 2b) is used as Raman signal is dominated by the material volume^3^,^4^.

Surprisingly, there is no change of the diamond peak maximum position including FWHM in dependence on the air annealing time and related size reduction of DNDs down to 2 nm (volumetrically). Our results thus indicate that the shift to lower wavenumbers and asymmetric broadening of the DND diamond peak in comparison to bulk diamond may actually corresponds to 2 nm or even smaller single crystal diamond core or a scattering domain^41^ either in form of a monolithic DNDs or ~2 nm crystallites within the DNDs. This is in agreement with the finding that the shift and broadening of the Raman diamond peak regularly observed for DNDs do not correspond to the phonon confinement in 5 nm diamond nanocrystals as the HPHT NDs of similar size exhibited a much smaller shift and broadening^3^. The presence of smaller crystalline domains inside a DND particle is rather typical and often multiple twinning is observed^4^,^42^,^43^. However, other effects such as crystal defects, grain boundaries, lattice strain, and chemical heterogeneities may also contribute to the Raman peak broadening. Therefore we demonstrate here that, size determination from Raman spectra^4^,^44^ of such complex and defective nanomaterial such as DNDs is inherently inaccurate.

The second remarkable observation is the stable intensity of the diamond peak shoulder around 1250 cm^−1^. Raman spectra of 5 nm HPHT NDs made by grinding do not contain such a feature^3^. This feature thus most likely originates from a disordered sp^3^ phase^45^ which is typically found in dynamically synthesized nanodiamonds, such as DNDs^16^ or NDs produced by laser-assisted techniques^46^. The disordered sp^3^ phase may be part of the sp^3^/sp^2^ transient layer which is believed to surround the diamond core of the DNDs^15^. It arises as a near surface stress-releasing layer due to various structural defects and the often irregular shape of DNDs (Supplementary Fig. S4) in contrast to well faceted HPHT NDs on the same size scale^3^. At the same time, it may occur also in the form of standalone disordered particles (Supplementary Fig. S5). Our Raman data indicate that the volume of the disordered sp^3^ phase remains constant as DNDs decrease in size, *i.e.* this layer/fraction must be persistent during the oxidative etching process and cannot be removed from DNDs by air annealing. It was shown by Oswald *et al*.^47^ that this feature reduces its intensity by selective removal of the smallest DNDs. This is confirmed also by our Raman data of non-centrifuged samples (see Supplementary Fig. S6), again due to the gradual volumetric decrease of the smallest DND fraction and the corresponding decrease of their Raman cross section.

The third characteristic Raman feature is the 1500–1800 cm^−1^ band, further denoted as the G band. It probably originates from a surface or near-surface sp^2^ carbon atoms and it combines the G-mode at 1582 cm^−1^ due to in-plane stretching in graphene, the D´ band at ∼1620 cm^−1^ caused by intravalley double resonance scattering in defective graphene^48^,^49^, and other sp^2^ forms like “isolated” dumbbell defects^50^ or sp^2^ chains since they have similar wavenumbers. Intensity of the G band follows a non-monotonic trend. At first, a slight increase in intensity occurs after annealing for 10 min. Then a gradual decrease is observed for longer annealing times (25–100 min). We suppose that this trend is due to an increase in the surface-to-volume ratio due the disintegration of the core agglutinates and the size reduction of DNDs on the one hand and on the other hand due to partially selective removal of the sp^2^ carbon atoms during the size reduction. In order to subtract the influence of the surface to volume ratio on the G band intensity we can use accurate AFM size analysis to obtain the relative intensity of the G band. These data are shown in the supplementary information (Fig. S7). From the presented size distribution data and the Raman spectra we determine the 25 min annealing as optimal for the highest yield of the smallest DNDs, while annealing for 50 min may be optimal in terms of higher DND purity.

It should be noted that the microscopic structural details on single particle level cannot be evaluated from the Raman spectra since the Raman spectroscopy provides averaged results. Therefore, it is useful to correlate the evolution of the Raman spectra with the STEM imaging of individual DNDs.

**Figure 5:**
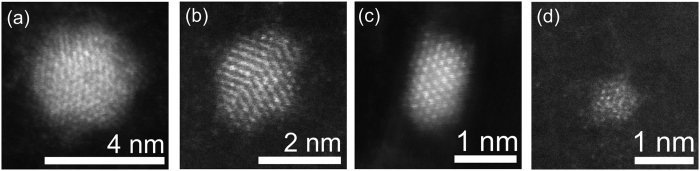
Scanning transmission electron microscopy images of the size reduced DNDs. STEM images of as received DNDs (**a**) and DNDs after annealing at 520 °C, 25 min showing various sizes from 4 to 1 nm (**b**–**d**).

Representative STEM images of the as received and annealed DNDs (520 °C, 25 min) are shown in Fig. 5. By analysis of the STEM images we identified a dependence of the DND structure on size and most probably also on the initial structure of a particular DND. In the sub-2 nm size range we observed not only the perfectly crystalline DNDs which are shown in Fig. 5b,c but also some number of partially disordered DNDs with crystalline domains. In the 1 nm range we observed particles with a hint of crystalline structure (Fig. 5d) or obviously amorphous DNDs. We did not find any perfectly crystalline DND below 1 nm by STEM.

In fact, the as-received DNDs already contain some amorphous particles (Supplementary Fig. S5). Both the as-received and annealed DNDs look similar and there is also no apparent graphitic shell around the DNDs which is regularly observed in the HRTEM images of DNDs^42^,^43^,^51^. This is consistent with the Raman data as the air annealing of the DNDs at 520 °C for 10 and 25 min results only in minor changes of the 1500–1800 cm^−1^ band. Therefore, the as-received DNDs may be considered as already very pure DNDs with a negligible amount of non-diamond carbon content removable by standard industrial cleaning processes. The stability limit of air-annealed HPHT NDs is around 1 nm since perfectly crystalline HPHT NDs were found down to 1.1 nm^3^. Here we show that the air annealing of DNDs at 520 °C for 25 min leads to maximal decrease in the mean DNDs size down to 1.4 nm and the mean size remains below 2 nm for 50, 75 and 100 minutes of annealing at 520 °C. Thus in the case of DNDs the stability limit is roughly estimated to be between 1–2 nm but it is certainly influenced by the complex structure and heterogeneity of the DNDs as shown here and reported recently^52^. Nevertheless, we suppose that if an initial DND particle is perfectly crystalline then it keeps its crystalline diamond structure down to 1 nm similarly to HPHT NDs^3^. An example of such a particle is shown in the Fig. 5c. Electron energy loss spectroscopy (EELS) analysis confirmed the imaged particles as nanodiamonds (Supplementary Fig. S8).

Based on the Raman and STEM data we suppose that the size decrease of an average single 4.5 nm particle proceeds as follows: sp^2^ defects (G band) are preferentially etched by oxygen during the annealing as they are more easily attacked by air oxygen than sp^3^ carbon^53^. The amorphous sp^3^ carbon content is constant (1250 cm^−1^ shoulder), *i.e.* persistent during the etching. Thus as the size of a DND decreases the ordered diamond core is turning to a disordered sp^3^ and eventually to amorphous sp^3^/sp^2^ particle at sizes below 1 nm, which is the final state of the annealing process before it is completely burned away. We suppose that the amorphization of DNDs is caused by surface induced stress, which increases with decreasing diameter of the DNDs due to the increase in the surface-to-volume ratio. It is also clear that the amorphous sp^3^/sp^2^ content occurs in the form of standalone amorphous particles or amorphous chunks attached to a diamond core. Therefore, the broadly used modeling of the structure of DNDs by core-shell models seems to be a very coarse approximation. DNDs might be instead considered as a heterogeneous mixture of particles ranging from perfect diamond nanocrystals to amorphous (nano)diamond-like particles.

## Conclusions

We have presented a method of controllable mean size reduction of DND powder from 4.6 nm down to 1.4 nm by using annealing in air for 25 min. In comparison with previous reports it turned out that the determination of a suitable annealing temperature is crucial for this effect. The optimal temperature of 520 °C for our DND powder was determined by TGA. At this temperature, the effects of the controllable size reduction of DNDs and enhanced disintegration of core agglutinates into individual DNDs occur at the same time. We used ultrasonication and centrifugation processes to obtain individual DNDs well dispersed in colloidal solutions. AFM and DLS revealed that the annealing in air leads predominantly to the mean size reduction down to 1.4 nm (number distribution) without noticeable particle loss. Annealing longer than 25 min leads to gradual particle loss, nevertheless, the DND mean size remains below 2 nm for up to 100 min of annealing. UV Raman spectroscopy and STEM confirmed the diamond structure of such molecular-sized DNDs. Surprisingly, in Raman the diamond peak did not change shape with the decreasing size of DNDs. Therefore, the broadened and shifted Raman diamond peak routinely observed in DNDs should be attributed to ~2 nm or even smaller scattering domains despite the typical 5 nm mean size of DNDs. Raman spectroscopy also showed that an amorphous sp^3^ phase is persistent during the annealing process, most likely due to strain inherently present in DNDs. STEM revealed that DNDs turn amorphous below 1 nm, possibly again due to strain. Overall, the presented results show that annealing in air can be used as a simple, efficient, controllable, high-yield, and easily scalable technique to obtain DNDs with sizes below 2 nm, narrow size distributions (less than ±1 nm), and a fully nanodiamond character. This opens new possibilities for fundamental and application studies of molecular-sized nanodiamonds.

## Methods

We used commercially available DNDs (distributor: New Metals and Chemicals Corp. Ltd., Kyobashi, manufacturer: Lingyun Granda Nano (China)) with a nominal size of 5 nm. The DNDs are made from TNT (trinitrotoluene) and RDX (hexogen) charge, cooled by a surrounding water shell. The DNDs were used in the as-received state (DND-as rec.) as a reference. The air annealing was performed in a laboratory furnace LV 15 (LAC) under an ambient air atmosphere at 520 °C for 10, 25, 50, 75, and 100 min. All colloidal solutions were prepared from 3 mg of the as-received and annealed DNDs by adding 2 mL of deionized water. The solutions were then ultrasonicated by means of an ultrasound horn (Hielscher) at 120 W for 1 hour to ensure a proper dispersion and then centrifuged (Eppendorf Mini plus) at 14000 rpm (13124 × g) for 1 h. After this treatment 1 mL of supernatant was carefully separated by a micropipette. For AFM size analysis the DNDs were deposited on Si substrates by immersing the Si substrates into the colloidal solution and sonicating in an ultrasonic bath for 10 min.

AFM was measured by an NTegra prima (NT-MDT) using Tap300Al-G cantilevers (Budgetsensors) with a nominal resonant frequency of 300 kHz and a tip radius below 10 nm that ensured sufficient lateral resolution to distinguish single particles. The amplitude of the tip oscillation was kept at 2 nm, setpoint was about 80%, *i.e.* the AFM measurements were performed in an attractive force AFM regime, as evidenced by a positive phase shift. The sizes of the nanoparticles were determined from the maximum particle height to exclude the effect of the AFM tip convolution. The mean height of the substrate was subtracted from the maximum particle height to obtain the size of the particle. The full width at half maximum (FWHM) of the substrate height histogram was always below 1 nm. The systematic error of the AFM size data has been thus estimated to ±0.5 nm or smaller. The resolution of the 1 × 1 μm^2^ scans was 512 × 512 pixels. This corresponds to the 1.95 nm of the scanner step. A typical 2 nm particle was imaged having a diameter of 13 nm and approximately 6 pixels were recorded on such particle per line providing reasonable accuracy. The particle size analysis was performed by the AFM software (Nova) and at least three randomly chosen spots were analyzed on each sample. Typical numbers of particles used for the particle size analysis was within 1000–6000, specifically: DND-as rec. 2755, DND-10: 4877, DND-25: 5263, DND-50: 4076, DND-75: 1527, DND-100: 1069.

DLS measurements of colloidal solutions were performed on a Malvern instrument Zetasizer Nano ZS equipped with a helium-neon laser (633 nm); the scattering angle was 173°. The refractive index of bulk diamond (2.4), viscosity of pure water (1.0020 mPa.s) were used to convert the measured intensity/size distributions to number/size and volume/size distributions. Each sample was analyzed by 5 subsequent runs and a typical DLS curve in between the extremes was chosen as characteristic for particular sample.

DNDs were further characterized by micro-Raman spectroscopy. Raman spectra were acquired using a Renishaw InVia Raman micro-spectrometer with a UV excitation wavelength of 325 nm from a He-Cd laser. The intensity of the incident laser was below 1 mW in order to minimize possible heat-induced changes of the samples. A volume of 20 μL of the colloidal solution was applied on a Si substrate by drop-casting and dried at 100 °C for 2 min in order to evaporate the water. All measurements were performed with a 40x objective with a numerical aperture of 0.5, resulting in a laser spot diameter of 20 μm. The accumulation time was set to 100 s to increase the signal to noise ratio. A spectral calibration was done on a bulk monocrystalline diamond sample. All the spectra were baseline corrected and normalized to diamond peak.

Surface chemistry of the annealed DNDs was studied by grazing angle reflectance (GAR) Fourier transform infrared (FTIR) spectroscopy. We used a nitrogen purged Thermo Nicolet8700 spectrometer equipped with the KBr beam splitter and mercury-cadmium telluride detector cooled by liquid nitrogen. The colloidal solutions (120 μL) were applied on the Au mirrors which were first oxidized in radio frequency plasma (45 W, 1 min) to achieve hydrophilic surfaces and thereby good spreading and adhesion of DNDs. The samples were dried at 100 °C for 2 min in order to evaporate bulk water from the Au surface. The spectra represent an average of 128 scans recorded with a resolution of 4 cm^−1^.

A TEM grid covered with CVD-grown graphene (Graphenea) was dipped into colloidal solution of DNDs and then rinsed in isopropanol. In order to avoid damage to the graphene support caused by surface tension during drying, a critical point drying technique was used. STEM imaging was carried out with a Nion UltraSTEM 100 operated at 60 kV which is well below knock on damage threshold for graphene^54^ as well as for DNDs^43^, and in ultrahigh vacuum. The achieved structural resolution was 1 Å.

## Additional Information

**How to cite this article**: Stehlik, S. *et al*. High-yield fabrication and properties of 1.4 nm nanodiamonds with narrow size distribution. *Sci. Rep.* **6**, 38419; doi: 10.1038/srep38419 (2016).

**Publisher's note:** Springer Nature remains neutral with regard to jurisdictional claims in published maps and institutional affiliations.

## Supplementary Material

Supplementary Information
