# Supplementary material for: High-yield fabrication and properties of 1.4 nm nanodiamonds with narrow size distribution
Source: Sci Rep. 2016 Dec 2;6:38419. doi: 10.1038/srep38419 (PMC5133551; doi:10.1038/srep38419)
Supplement: Supplementary Information [file srep38419-s1.doc]

**SUPPORTING INFORMATION**

“High-yield fabrication and properties of 1.4 nm nanodiamonds with narrow size distribution”

Stepan Stehlik^*1^, Marian Varga^1^, Martin Ledinsky^1^, Daria Miliaieva^1,2^, Halyna Kozak^1^, Viera Skakalova^3^, Clemens Mangler^3^, Timothy J. Pennycook^3^, Jannik C. Meyer^3^, Alexander Kromka^1^, Bohuslav Rezek^1,2^

*^1^ Institute of Physics ASCR, Cukrovarnická 10, 162 00 Prague 6, Czech Republic*

*^2^ Faculty of Electrical Engineering, Czech Technical University in Prague, Technická 2, 16627 Prague 6, Czech Republic*

*^3^ Physics of Nanostructured Materials, Faculty of Physics, University of Vienna, Boltzmanngasse 5, 1090 Vienna, Austria*

e-mail: [stehlik@fzu.cz](mailto:stehlik@fzu.cz)

Figure S1 shows the relative loss of DNDs mass during annealing at 520°C as a function of time for up to 100 min. The dependence is well fitted by an exponential decay which yields etching time constant of 22±3 min.


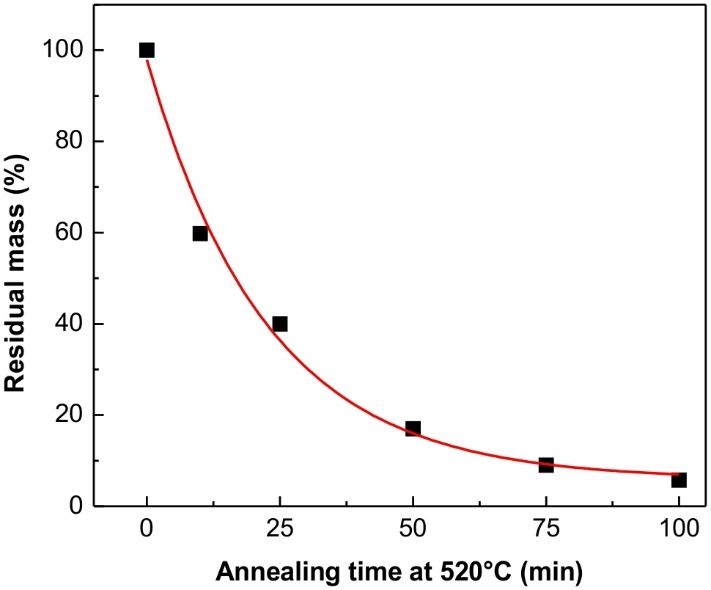


**Figure S1.** Weight loss dependence of the DNDs on the annealing time at 520°C (black) fitted by an exponential decay function (red).


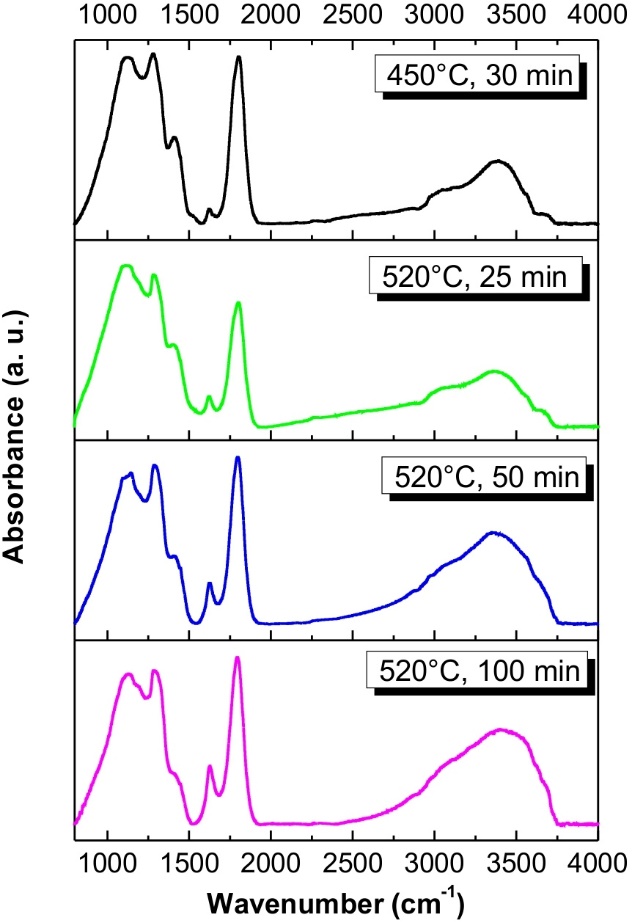


**Figure S2.** FTIR spectra of surface oxidized DNDs (450°C, 30 min, black) and of size reduced DNDs after air annealing at 520°C for 25 min (green), 50 min (blue), and 100 min (magenta).

Figure S2 shows FTIR spectra of only surface oxidized DNDs which were annealed in air at 450°C for 30 min^1^ and size reduced DNDs after air annealing at 520°C for up to 100 min. It is obvious from the spectra that there is no significant change of the DND surface chemistry after the size reduction. All the spectra are dominated by the oxygen containing surface functional groups giving rise to O–H stretching (2500-3500 cm^-1^) and bending (1625 cm^-1^) vibrations from water adsorbed on the DND surface^2^ or surface functional groups^3^. We further identified the C=O stretching at 1800 cm^-1^ which indicates the presence of anhydrides and lactones which shift the carbonyl peak to higher wavenumbers than in the carboxyl group. A broad absorption feature at 800–1500 cm^-1^ may include, besides C–O–C stretching, the O–H deformation, C–C stretching vibrations, peaks related to nitrogen defects, and other groups^4^. As oxygen is a highly electronegative element it forms strong dipoles with other less electronegative elements such as C or H. Therefore the presence of these surface oxygen containing groups ensures good solubility of the air annealed DNDs in polar solvents such as water through the formation of hydrogen bonds.


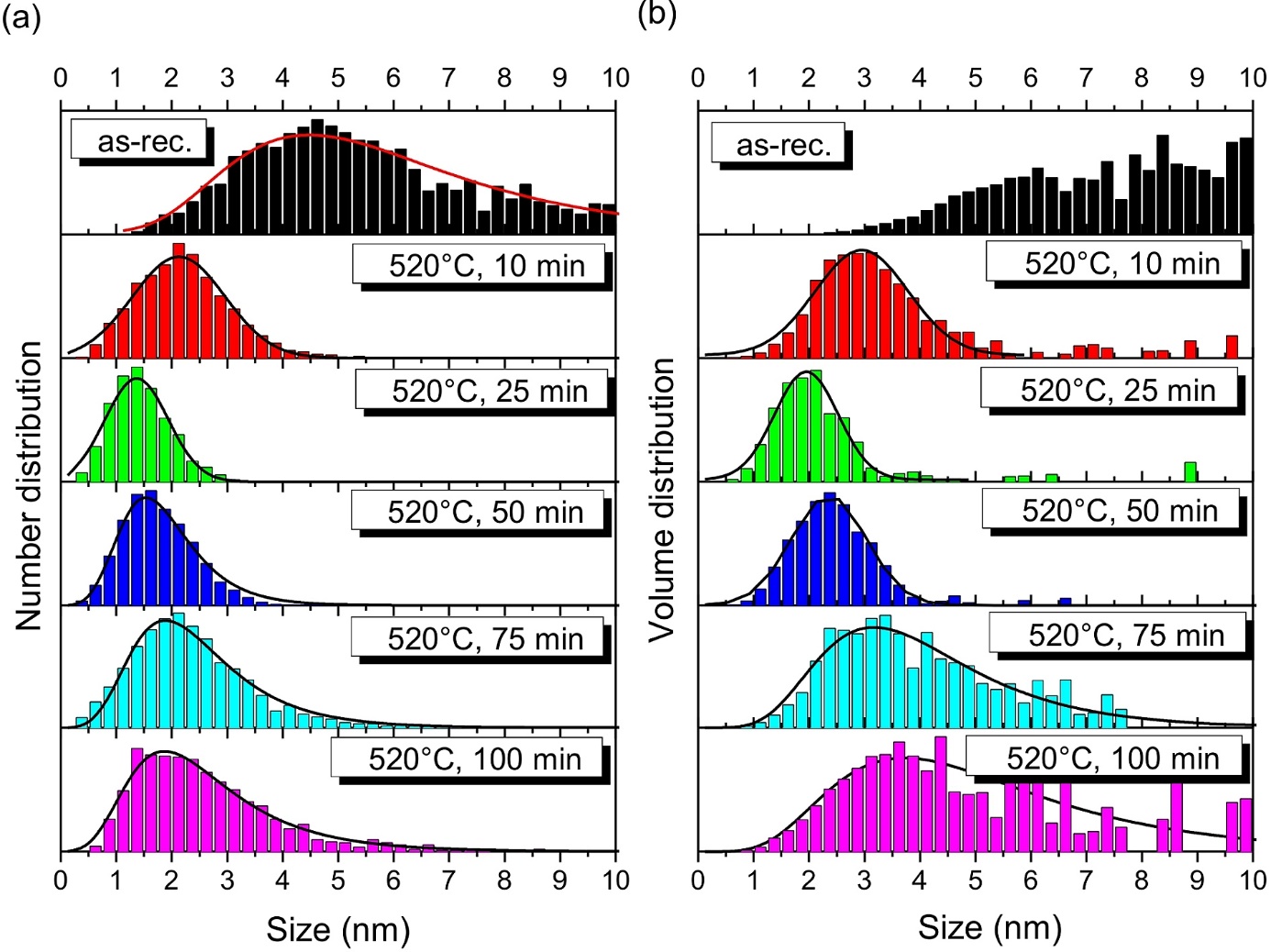


**Figure S3**. Size distribution histograms derived from the AFM images of the as-received DNDs and the DNDs air-annealed at 520°C for 10 min, 25 min, 50 min, 75 min, and 100 min plotted as (a) number distribution (a) and volume distribution (b).


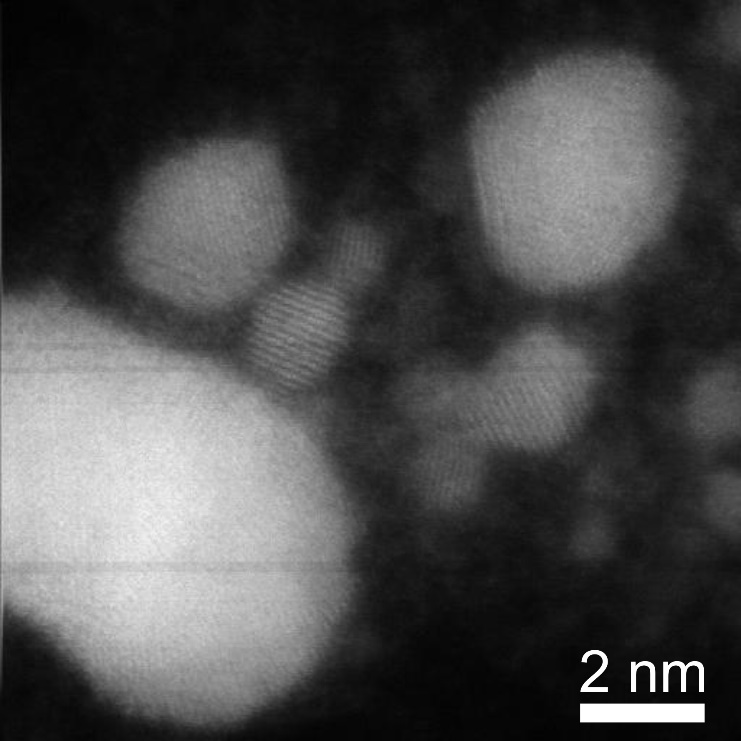


**Figure S4**. STEM image of a large ~ 9 nm DND particle in the as-received DND sample.

Figure S4 shows a STEM image of a large DND particle (~ 9 nm) of irregular shape that was identified in the as-received DND sample. Presence of such a large DND particle, considerably deviating from the mean size of 4.6 nm is not surprising since DNDs up to 30 nm^5^ were found in a commercial powder with a similar Raman spectral character, i.e. structure, to ours. Due to their large volumes, such particles may significantly contribute to the nanodiamond mass and Raman signal despite their relatively low number.


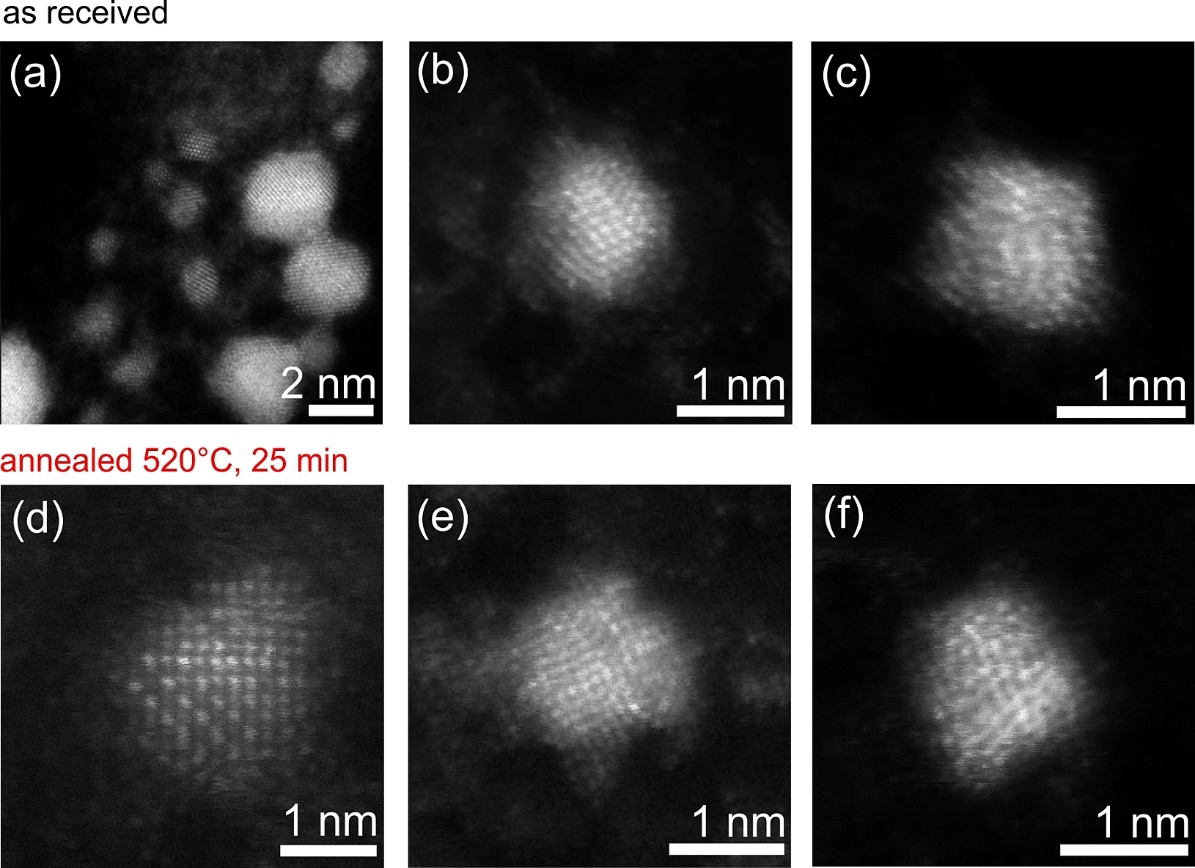


**Figure S5.** Comparison of STEM images of as-received and air annealed (520°C, 25 min) DNDs. No visual difference is apparent, i.e. the DND diamond structure is not degraded by the oxidative etching. (c) and (f) shows presence of disordered/amorphous DNDs in both as-received as well as annealed samples.

Typical STEM images of as-received DNDs of various sizes are shown in Fig. S5a-c. Crystalline (1.5 nm) as well as disordered DNDs (1.1 nm) are shown in the Figure S5b and c. This demonstrates that a certain number of very small as well as amorphous DNDs are already present in the initial as-received DND powder. Note that no obvious sp^2^-like shells, frequently seen in TEM images of DNDs^6–9^, are observed in any of the STEM images. Figures S5d-f show DNDs after the annealing treatment at 520°C, 25 min, i.e. these DNDs have a decreased size. Figure S5d, shows a fully crystalline 2 nm DND which consists of a single diamond crystal. The particle in Figure S5e appears in contrast as a polycrystal with sub nm crystallites. Such polycrystallinity is common for DNDs^7,9^. Figure S5f exhibit a nearly amorphous structure. The STEM images in Figure S5 demonstrate that the air annealing at 520°C does not have any negative impact on the diamond structure of DNDs and that it can be used for the size reduction of DNDs without any unwanted structural changes or DND transformations such as graphitization^10^.


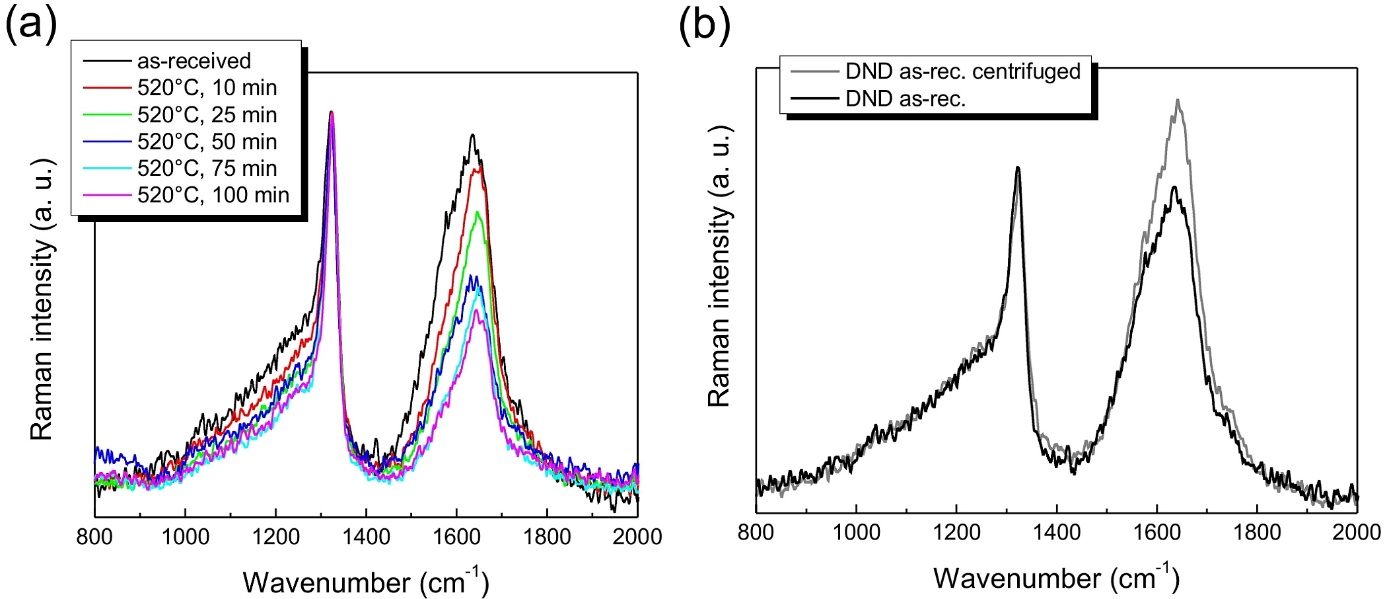


**Figure S6.** Evolution of Raman spectra of non-centrifuged DNDs before (as-received) and after air annealing at 520°C for different times (a). Comparison of Raman spectra of non-centrifuged DNDs and centrifuged as-received DNDs (b).

Figure S6a shows the Raman spectra evolution of non-centrifuged samples, i.e. with the full particle distribution after the air annealing at 520°C. We suppose that both 1200 cm^-1^ and 1500-1800 cm^-1^ features decrease intensity due to a continuous decrease of the smallest DND volumetric contribution to the total Raman signal. This effect must of course involve the removal of the size reduced DNDs after annealing times longer than 25 min. See the main text for more details. The evolution of the diamond peak and related low frequency shoulder is very similar to that reported by Osswald et al.^5^ Figure S6b shows a considerable difference in the G band intensity of the as-received DNDs prior to centrifugation (black) and after centrifugation (grey). In principle, the Raman spectra of centrifuged and non-centrifuged as-received DNDs should not differ from each other since the mean size of DNDs is in both cases still 4.5 nm. This assumption is verified by the perfect match of the diamond peak and its low frequency shoulder in both samples. However after sonication and centrifugation the DNDs taken from the supernatant are mostly no longer embedded in the core agglutinates (see the AFM distribution in the main text). Then the higher intensity of the G-band in the centrifuged sample may be ascribed to specific sp^2^ structures such the sp^2^ chains which arise due to increase of the specific surface of the released DNDs from the core agglutinates.


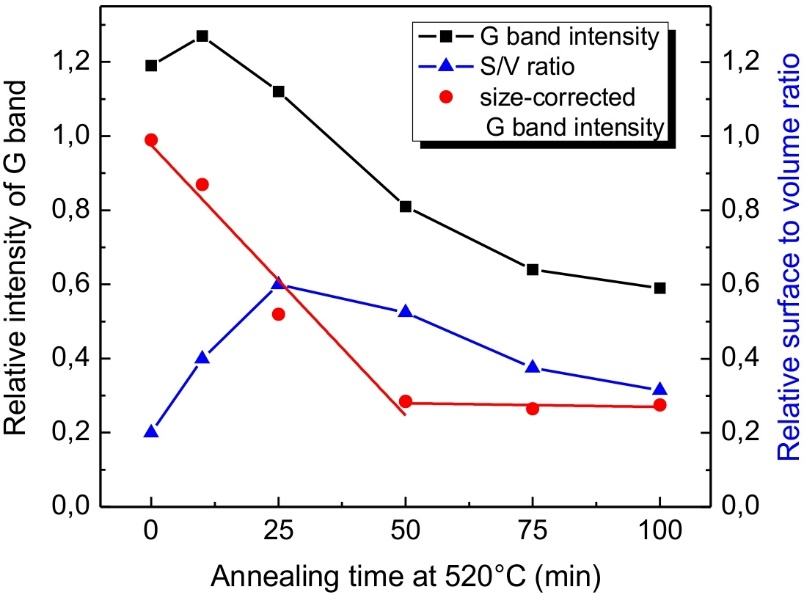


**Figure S7.** Relative intensity of the G band (black squares), relative surface to volume ratio (blue triangles) and size-corrected evolution of G band intensity (red circles) with schematically indicated decrease and plateau regions.

From the analysis of the intensity of the G band from Fig. 5 in the main article we found a non-monotonic trend. The dependence on the annealing time at 520°C of the G band relative maxima is shown in the Fig. S7 (black). First, a slight increase of its intensity after annealing occurs at 10 min, then a gradual decrease is observed for longer annealing times at 520°C (25-100 min). In order to subtract the influence of the surface to volume (S/V) ratio on the G band intensity we can use accurate AFM size analysis. Specifically, we used mean size values from the volumetric distributions (Fig. 2b) and a normalization factor of 1/40. Such recalculated values are shown in the graph (blue). When we subtract the S/V ratio from the overall G band trend we obtain a “size-corrected evolution of the G band intensity” curve with two linear regions. These data are shown in red in the Figure S7. Initially it seems there is a linear trend up to 50 minutes that likely corresponds to constant etch rate of the surface sp^2^ phase. From 50 minutes onward there is a plateau region which indicates that sp^2^ phase content is constant and cannot be etched any more.


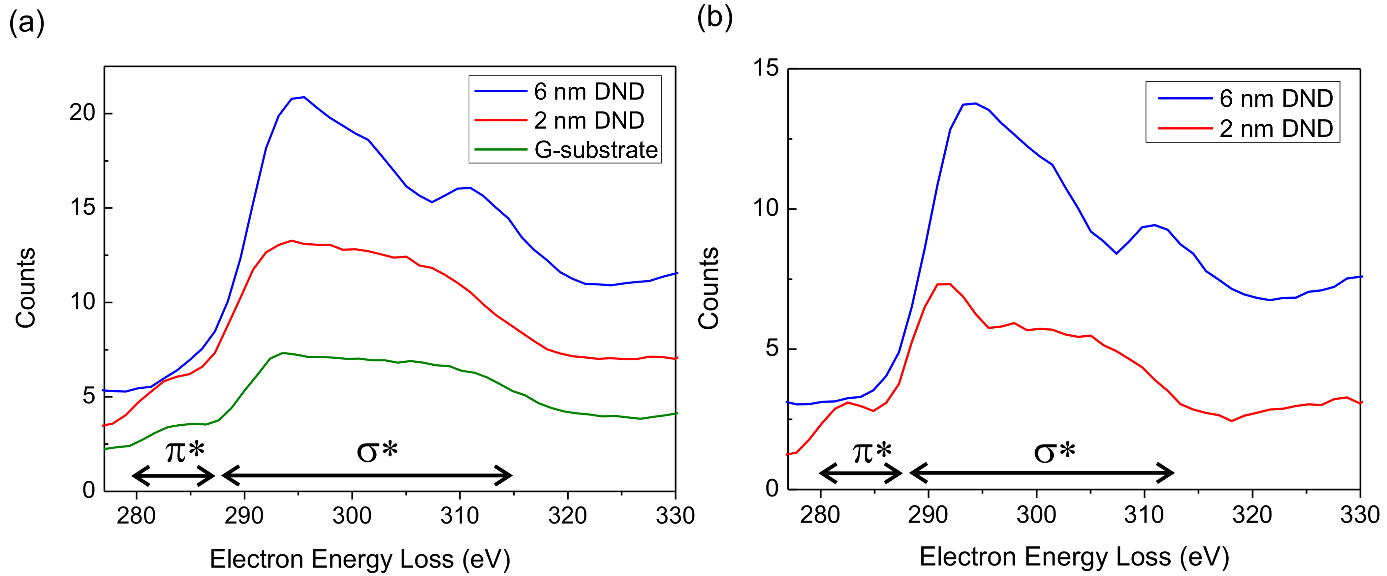


**Figure S8**. Raw EELS spectra of a 6 nm DND particle (blue), a 2 nm DND particle (red) and graphene substrate (green) (a). EELS spectra of the DND particles after subtraction of the graphene support contribution.

Electron energy loss spectroscopy (EELS) was performed in an UltraSTEM 100. With the ability to collect spectra at atomic spatial resolution, the UltraSTEM enables one to spectroscopically examine individual nanoparticles^11^. Figure S8a shows raw EELS spectra of typical 6 nm (blue) and 2 nm (red) DNDs after air annealing at 520°C for 25 minutes. The DNDs were deposited on a graphene support (green). The spectra were all acquired with the same exposure time of 0.85 seconds, and in order to reveal the EELS signal coming only from the DNDs we subtracted the EELS signal of the graphene support from the spectra of DNDs (see Figure S8b). The graphene-subtracted EELS spectra of both the 6 nm and 2 nm DNDs exhibit a 1s-σ* transition feature starting at approximately 290 eV that is characteristic of carbon in a sp^3^ coordination. The spectrum from the 6 nm DND is typical for nanodiamond, however the 2 nm DND shows an additional peak at 283 eV corresponding to 1s-π* transitions that is associated with carbon in a lower (e.g. sp^2^) or distorted sp^3^ coordination^12^. This most probably relates with higher surface-to-volume ratio of the 2 nm DND in comparison with 6 nm DND, i.e. the sp^2^ or distorted sp^3^ carbon atoms are located on the surface or in the near surface region^12^. This conclusion is supported by the Raman spectra shown and discussed in the main text.

**References**

1. Kozak, H. *et al.* Chemical modifications and stability of diamond nanoparticles resolved by infrared spectroscopy and Kelvin force microscopy. *J. Nanoparticle Res.* **15,** (2013).

2. Stehlik, S. *et al.* Water interaction with hydrogenated and oxidized detonation nanodiamonds — Microscopic and spectroscopic analyses. *Diam. Relat. Mater.* **63,** 97–102 (2016).

3. Kozak, H. *et al.* Oxidation and reduction of nanodiamond particles in colloidal solutions by laser irradiation or radio-frequency plasma treatment. *Vib. Spectrosc.* **83,** 108–114 (2016).

4. Stehlik, S. *et al.* Size and Purity Control of HPHT Nanodiamonds down to 1 nm. *J. Phys. Chem. C* **119,** 27708–27720 (2015).

5. Osswald, S., Havel, M., Mochalin, V., Yushin, G. & Gogotsi, Y. Increase of nanodiamond crystal size by selective oxidation. *Diam. Relat. Mater.* **17,** 1122–1126 (2008).

6. Pichot, V., Comet, M., Risse, B. & Spitzer, D. Detonation of nanosized explosive: New mechanistic model for nanodiamond formation. *Diam. Relat. Mater.* **54,** 59–63 (2015).

7. Iakoubovskii, K., Mitsuishi, K. & Furuya, K. High-resolution electron microscopy of detonation nanodiamond. *Nanotechnology* **19,** 155705 (2008).

8. Arnault, J.-C. *et al.* Surface chemical modifications and surface reactivity of nanodiamonds hydrogenated by CVD plasma. *Phys. Chem. Chem. Phys.* **13,** 11481 (2011).

9. Williams, O. A. *et al.* Size-Dependent Reactivity of Diamond Nanoparticles. *ACS Nano* **4,** 4824–4830 (2010).

10. Mykhaylyk, O. O., Solonin, Y. M., Batchelder, D. N. & Brydson, R. Transformation of nanodiamond into carbon onions: A comparative study by high-resolution transmission electron microscopy, electron energy-loss spectroscopy, x-ray diffraction, small-angle x-ray scattering, and ultraviolet Raman spectroscopy. *J. Appl. Phys.* **97,** 74302 (2005).

11. Pichot, V. *et al.* High nitrogen doping of detonation nanodiamonds. *J. Phys. Chem. C* **114,** 10082–10087 (2010).

12. Turner, S. *et al.* Aberration-corrected microscopy and spectroscopy analysis of pristine, nitrogen containing detonation nanodiamond: Microscopy and spectroscopy analysis of pristine, nitrogen containing DND. *Phys. Status Solidi A* **210,** 1976–1984 (2013).
